# FHL2 fused with biotin ligase shuttles between focal adhesions and the nucleus in a myosin II-dependent manner

**DOI:** 10.17912/micropub.biology.001669

**Published:** 2025-08-28

**Authors:** Yukari Fujimoto, Masaya Fujimoto, Daijiro Konno, Naotaka Nakazawa

**Affiliations:** 1 Graduate School of Science and Engineering, Kindai University, Higashiosaka-city, Osaka, Japan; 2 Faculty of Science and Engineering, Kindai University, Higashiosaka-city, Osaka, Japan

## Abstract

Four-and-a-half LIM domains 2 (FHL2) is a molecule that plays a key role in cell proliferation in response to mechanical signals. It shuttles between focal adhesions (FAs) or stress fibers (SFs) and the nucleus in a force-dependent manner. FHL2 interacts with other cytoskeletal molecules at FAs and SFs, but FHL2 interacts with transcriptional factors in the nucleus. This leads to modulation of gene expression for cell proliferation. However, the overall picture of interacting proteins with FHL2 at different regions is poorly understood. Here, we report a stable cell line that expresses FHL2-GFP-BirA, a fusion protein of FHL2 with biotin ligase. FHL2-GFP-BirA localizes at the FAs but accumulates in the nucleus after myosin II inhibition, exhibiting behavior similar to endogenous FHL2. These results suggest that the BioID technique can be used to identify proteins interacting with FHL2 under different mechanical conditions.

**Figure 1. FHL2-GFP-BirA shuttles between FAs and the nucleus in Myosin II activity dependent manner:**
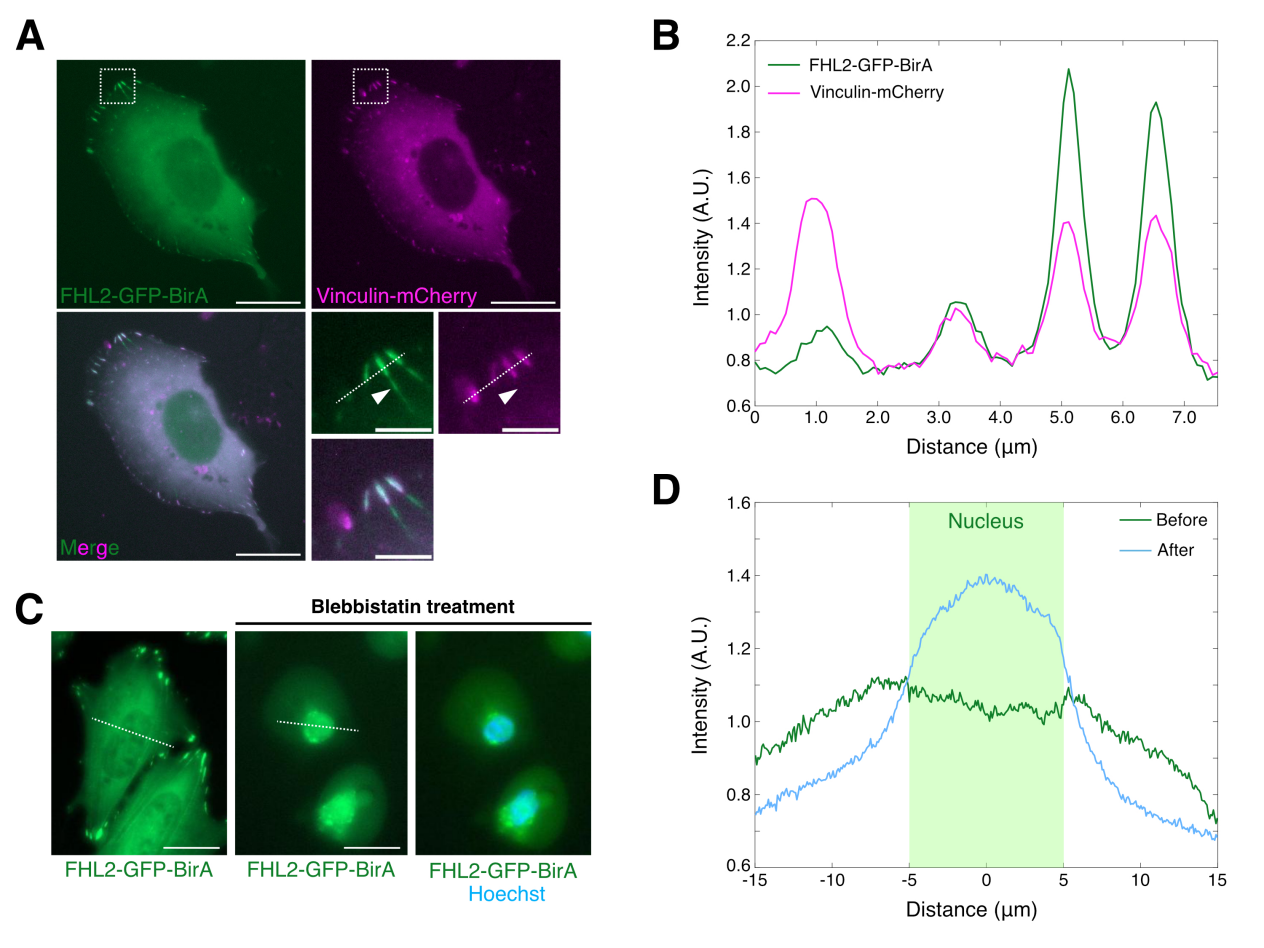
A: FHL2-GFP-BirA localization (top left, green), Vinculin-mCherry localization (top right, magenta) in U2OS cell. Merged image of top images is shown at left bottom. High magnified images of white box in top pictures are shown at right bottom. Scale bars: 20 μm (top left, top right, and bottom left), 10 μm (high magnified images). B: A graph showing the intensity profile of FHL2-GFP-BirA (line with green color) and Vinculin-mCherry (line with magenta color) on white dot line in Figure1A. C: FHL2-GFP-BirA localization (green) in U2OS cell with Blebbistatin treatment. The nucleus is visualized by hoechst staining (Blue). Scale bars: 20 μm (top left, top right, and bottom left). D: A graph showing the intensity profile of FHL2-GFP-BirA on white dot line in Figure1C (before Blebbistatin treatment; line with green color, after Blebbistatin treatment; line with blue color).

## Description

Cytoskeletal regulation plays a crucial role in several cellular functions, such as cell migration, cell differentiation, and cell proliferation (Vogel and Sheetz 2006; Vining and Mooney 2017; SenGupta, Parent, and Bear 2021 ). Cytoskeletal proteins are involved in these functions by facilitating the exertion of mechanical force. Additionally, some cytoskeletal proteins are activated by mechanical cues, which leads to activation of intracellular signaling pathways to modulate the expression of downstream genes. Thus, multiple roles of cytoskeletal proteins are critical for cellular functions.

Four-and-a-half LIM domains 2 (FHL2) is a cytoskeletal protein that plays multiple roles in a force-dependent manner (Fujimoto and Nakazawa 2024). FHL2 has a specific structure consisting of a tandem zinc-finger motif called the LIM domain. This motif was first discovered in the proteins, Lin11, Isl-1, and Mec-3. While the zinc-fingers generally bind directly to DNA, the LIM domain mediates protein-protein interactions (Kadrmas and Beckerle 2004; Anderson et al. 2021). FHL2 interacts with both cytoskeletal proteins and transcriptional factors by shuttling between the cytoplasm and the nucleus. This mediates intracellular signaling under the control of cytoskeletal regulators (Muller et al. 2002; Schiller et al. 2011). We previously reported that FHL2 translocates from the cytoskeletal structures to the nucleus in a manner that is dependent on the stiffness of the extracellular matrix and intracellular contractility (Nakazawa et al. 2016; Fujimoto and Nakazawa 2024). At the molecular level, phosphorylation of FHL2 by FAK is critical for its accumulation in the nucleus, leading to modulation of p21 gene expression. A recent study reported that direct binding to tense actin fibers depends on a conserved phenylalanine residue among several LIM domain proteins (Sun et al. 2020; Sun and Alushin 2023). Furthermore, specific amino acid residues in the LIM domain of FHL2 are critical for its nuclear accumulation in a force-dependent manner (Sun et al. 2020). Thus, FHL2 appears to interact with multiple proteins at the cytoskeletal structures in both the cytoplasm and the nucleus. However, the kind of proteins that interact with FHL2 in different locations remains elusive (Tran et al. 2016; Fujimoto and Nakazawa 2024).

BioID is a proximity labeling tool for target proteins (Choi-Rhee, Schulman, and Cronan 2004; Roux et al. 2012; Sears, May, and Roux 2019). Biotin ligase, an enzyme that biotinylates an interacting protein, is utilized to mark the interacting protein in this tool. Because the biotinylating of the interacting protein remains after dissociation, we can isolate the interacting proteins based on their history of association with the target protein (Guo et al. 2023). Recent studies using BioID have identified a new interacting partner of scaffolding proteins (Dong et al. 2016; Chastney et al. 2020; He et al. 2023). However, this technique has not yet been used to screen for the interacting partners of FHL2 in different mechanical environments.

Here, we report a new expression vector of FHL2-GFP-BirA gene (pPB-CAG-FHL2-GFP-BirA) for the BioID screen. In this vector, a hyperactive piggyBac transposase system has been combined to facilitate integration of the FHL2-GFP-BirA gene into the genome (Yusa et al. 2011; Fujita et al. 2020). Since FHL2 localizes at focal adhesions (FAs) and stress fibers (SFs) in the cell on rigid surfaces, we first checked the localization of FHL2-GFP-BirA in U2OS cells, a human osteosarcoma cell line, which were cultured on the glass surface (Nakazawa et al. 2016). The FAs in the cells were visualized based on Vinculin-mCherry localization. We confirmed that FHL2-GFP-BirA colocalized with Vinculin-mCherry ( Figure 1A ). Quantification of FHL2-GFP-BirA intensity profiling suggests that most FHL2-GFP-BirA signals overlap with the Vinculin-mCherry signal ( Figure 1B ). However, FHL2-GFP-BirA sometimes showed linear distribution from Vinculin-mCherry-positive FAs to the center direction (arrowhead in Figure 1A ). Because FHL2 localizes at both FAs and SFs, FHL2-GFP-BirA might accumulate at SFs in the cell on the rigid substrates (Nakazawa et al. 2016; Sun et al. 2020).

To investigate the localization of FHL2 in different mechanical conditions further, we examined the localization of FHL2-GFP-BirA in U2OS cells following the inhibition of myosin II activity. Previous studies have suggested that inhibiting myosin activity by blebbistatin induces FHL2 to localize at the nucleus (Schiller et al. 2011; Nakazawa et al. 2016; Sun et al. 2020). As shown in Figure 1C, the FHL2-GFP-BirA signal disappeared from the cell periphery following blebbistatin treatment. In contrast, the FHL2-GFP-BirA signal in the nucleus appeared to increase. Quantification of FHL2-GFP-BirA signal intensity indicates a clear increase ( Figure 1D ). These data suggest that inhibiting myosin II activity enhances FHL2-GFP-BirA localization to the nucleus.

In this study, we demonstrated the localization of FHL2 fused with biotin ligase (FHL2-GFP-BirA) within the cell on a glass surface. We found that inhibition of myosin II activity resulted in the nuclear localization of FHL2-GFP-BirA. Our results suggest that biotin ligase does not inhibit FHL2 localization at FAs and the nucleus in a force-dependent manner. This indicates that the BioID system could be useful for identifying the interacting partners of FHL2 under different force conditions. However, given that various types of biotin ligase have been reported, including TurboID and AirID, selecting the appropriate biotin ligase might be crucial for identifying the interacting partners of FHL2 (Guo et al. 2023; Fujimoto and Nakazawa 2024).

## Methods

**Plasmids:** To generate pPB-CAG-FHL2-GFP-BirA construct, FHL2-GFP cDNA (Nakazawa et al. 2016) and BirA cDNA (Guo et al. 2014) were recombined into the EcoRI site in pPB-LR5-Core2-CAG-HA-N1-Core2 plasmid vector using In-Fusion cloning (Takara) (Fujita et al. 2020). In terms of the pCAG-vinculin-mCherry constructs, mouse vinculin cDNA from a mouse brain cDNA library was recombined into pENTR1A. Recombination into pCAG-Dest-mCherry was performed by LR recombination with Gateway LR Clonase II Enzyme Mix (Invitrogen) as previously described (Nakazawa et al. 2025).

**Cell culture and transfections:** U2OS cell line was obtained from ECACC (EC92022711-F0). U2OS cells were cultured in McCoy’s 5A medium (Cytiva, SH30200.01) with 10% (v/v) FBS (Gibco) and 1% penicillin/streptomycin (Nacalai). A hyperactive piggyBac transposase system has been used to obtain stable cell line expressing FHL2-GFP-BirA (Yusa et al. 2011). pPB-CAG-FHL2-GFP-BirA and pCAX-hyPBase were transfected with NEPA21 Type II (Nepagene). After transfection, single cell with GFP expression was isolated. To observe co-localization of FHL2-GFP-BirA and Vinculin, pCAG-vinculin-mCherry was transfected with NEPA21 Type II (Nepagene).

**Cell imaging:** A glass bottom dish (Iwaki) was coated with Poly-D-Lysine (PDL) (Sigma-Aldrich, p6407) overnight. After PDL coating, the glass surface was coated with Fibronectin (Corning, 354008) overnight. After washing with PBS, cells were seeded. The cells were cultured for 12 hours before observation. Cell imaging has been done using Axio Observer 7 with a x63 lens (NA: 1.40) (Zeiss). The nucleus was visualized using Hoechst (Dojindo, H342) 30 minutes after para-amino-Blebbistatin treatment (50 μM, Cayman Chemical Company, 22699).

**Quantitative image analyses:** Image analyses were performed using Fiji (ImageJ 2.0), the ZEN application (Zeiss). Data were analyzed using MATLAB (MathWorks).

## Reagents

**Chemical reagents used in this study:**

In-Fusion HD Cloning Kit; TAKARA, 639648

EcoRI; FastGene, FG-EcoRI

LR Clonase II Enzyme Mix; ThermoFisher, 11791020

McCoy’s 5A medium; Cytiva, SH30200.01

Fetal Bovine Serum; Gibco, 10270106

Penicillin-Streptomycin; Nacalai tesque, 09367-34

Opti-MEM Reduced Serum Medium; ThermoFisher, 31985062

Poly-D-lysine hydrobromide (PDL); Sigma-Aldrich, p6407

Fibronectin, Human; Corning, 354008

Hoechst 33342 solution; Dojindo, H342

para-amino-Blebbistatin; Cayman Chemical Company, 22699

**A cell line used in this study:**

U2OS cell (Human osteosarcoma); ECACC, EC92022711-F0

**Plasmids used in this study:**

pCMV-FHL2-eGFP; Nakazawa et al., 2016

pJTI-E-cad- BirA; Guo et al., 2014

pPB-LR5-Core2-CAG-HA-N1-Core2; Fujita et al., 2020

pPB-CAG-FHL2-GFP-BirA; This study

pCAX-hyPBase; Fujita et al., 2020

pENTR1A; Nakazawa et al., 2016

pCAG-Dest-mCherry; Nakazawa et al., 2025

pCAG-vinculin-mCherry; This study
